# Supplementary material for: Illuminating Dark Proteins using Reactome Pathways
Source: bioRxiv. 2023 Jun 5:2023.06.05.543335. Preprint. [Version 1] doi: 10.1101/2023.06.05.543335 (PMC10274615; doi:10.1101/2023.06.05.543335)
Supplement: Supplement 1 [file NIHPP2023.06.05.543335v1-supplement-1.pdf]

bioRxiv preprint doi: https://doi.org/10.1101/2023.06.05.543335; this version posted June 5, 2023. The copyright holder for this preprint (which was not certified by peer review) is the author/funder, who has granted bioRxiv a license to display the preprint in perpetuity. It is made available under aCC-BY 4.0 International license.

Supplemental Figures

bioRxiv preprint doi: https://doi.org/10.1101/2023.06.05.543335; this version posted June 5, 2023. The copyright holder for this preprint (which was not certified by peer review) is the author/funder, who has granted bioRxiv a license to display the preprint in perpetuity. It is made available under aCC-BY 4.0 International license.

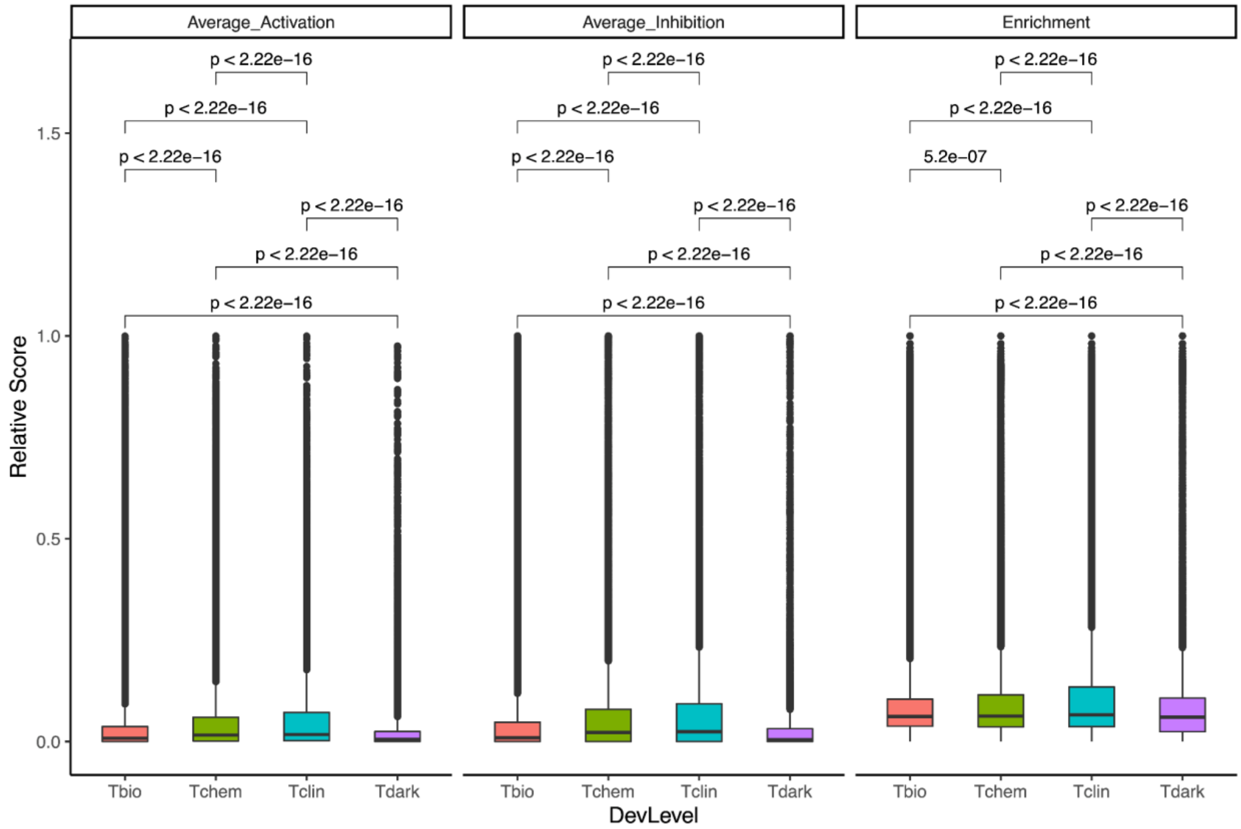

Figure S1. Box plot of interaction pathway scores for proteins categorized as Tbio, Tchem, Tclin, and Tdark. P-values were determined based on ANOVA.

bioRxiv preprint doi: https://doi.org/10.1101/2023.06.05.543335; this version posted June 5, 2023. The copyright holder for this preprint (which was not certified by peer review) is the author/funder, who has granted bioRxiv a license to display the preprint in perpetuity. It is made available under aCC-BY 4.0 International license.

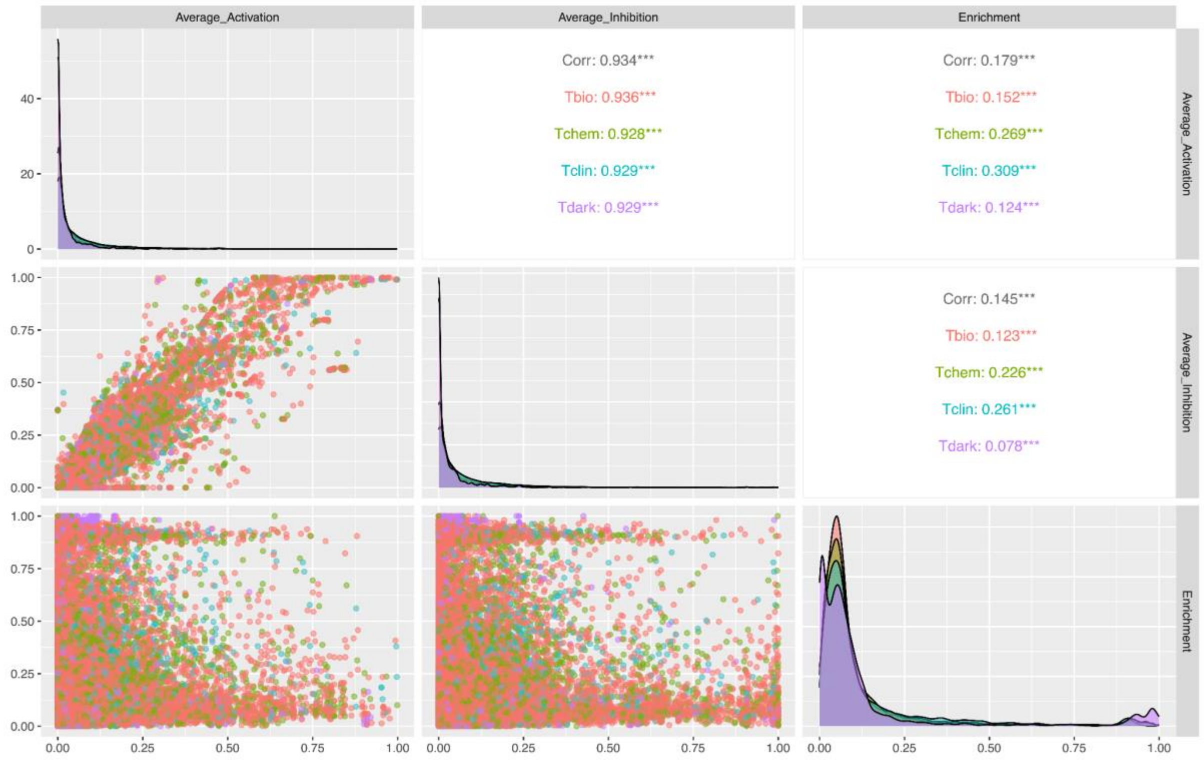

Figure S2. Correlation plot showing significant correlations between the three interacting pathway scores, Average_Activation, Average_Inhibtion and Enrichment. P-values are less than 0.001 based on 10% sampled data points.

bioRxiv preprint doi: https://doi.org/10.1101/2023.06.05.543335; this version posted June 5, 2023. The copyright holder for this preprint (which was not certified by peer review) is the author/funder, who has granted bioRxiv a license to display the preprint in perpetuity. It is made available under aCC-BY 4.0 International license.

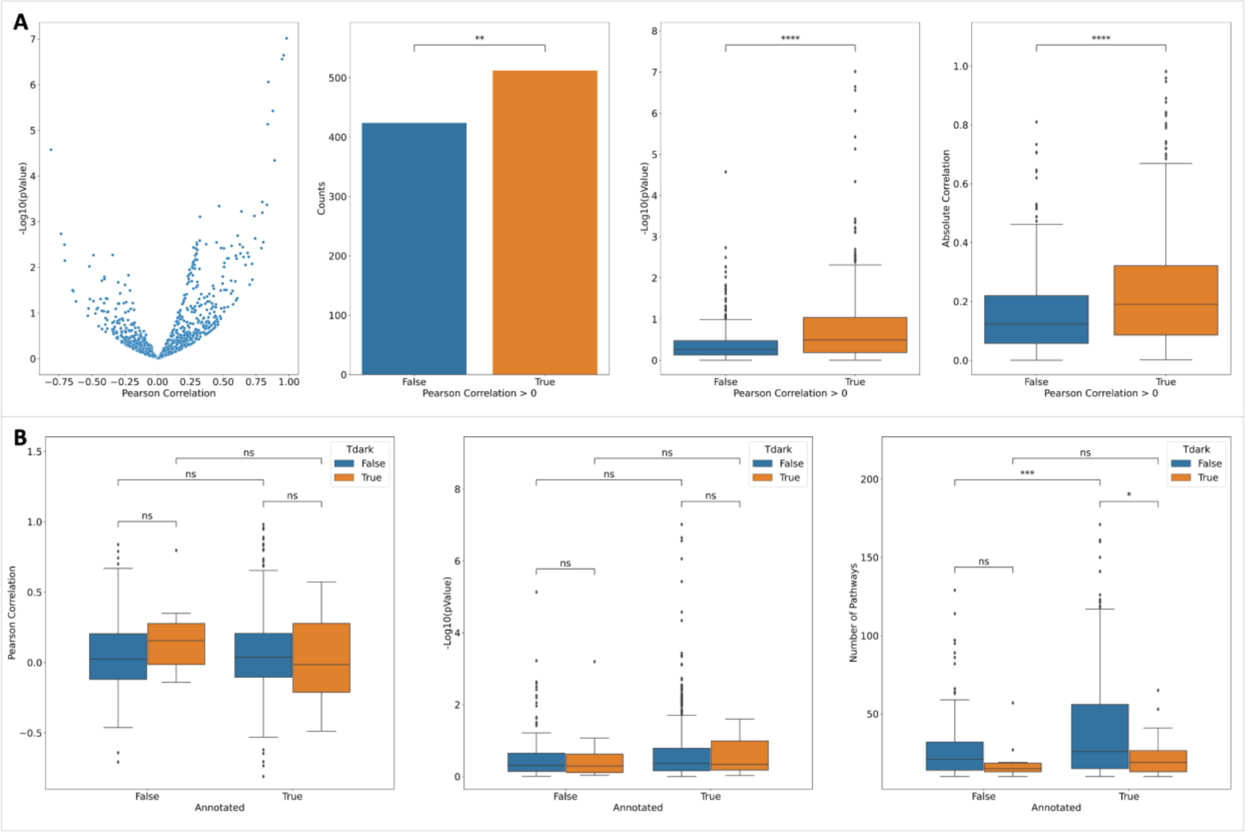

Figure S3. scRNA-seq analysis results support predicted interacting pathways by showing a significantly positively skewed distribution of correlations between average_activation_scores based on predicted FIs and enrichment score based on scRNA-seq coexpression (A) and unbiased distributions between annotated and not-annotated dark and not-dark proteins (B). The left-most panel in B shows the numbers of interacting pathways used for correlation calculation for individual proteins. The numbers of pathways used for correlation calculation between average_activation and enrichment score based on scRNA-seq co-expression are smaller than ones shown in Figure 4. This is because some proteins may functionally interact with proteins annotated in pathways but fail to have quantitative impact on pathway activities, according to our simulation approach. P-value: ****: <= 1.0E-04, ***: 1.00e-04 < p <= 1.00e-03, **: 1.00e-03 < p <= 1.00e-02, *: 1.00e-02 < p <= 5.00e-02, ns: p <= 1.00e+00.

bioRxiv preprint doi: https://doi.org/10.1101/2023.06.05.543335; this version posted June 5, 2023. The copyright holder for this preprint (which was not certified by peer review) is the author/funder, who has granted bioRxiv a license to display the preprint in perpetuity. It is made available under aCC-BY 4.0 International license.

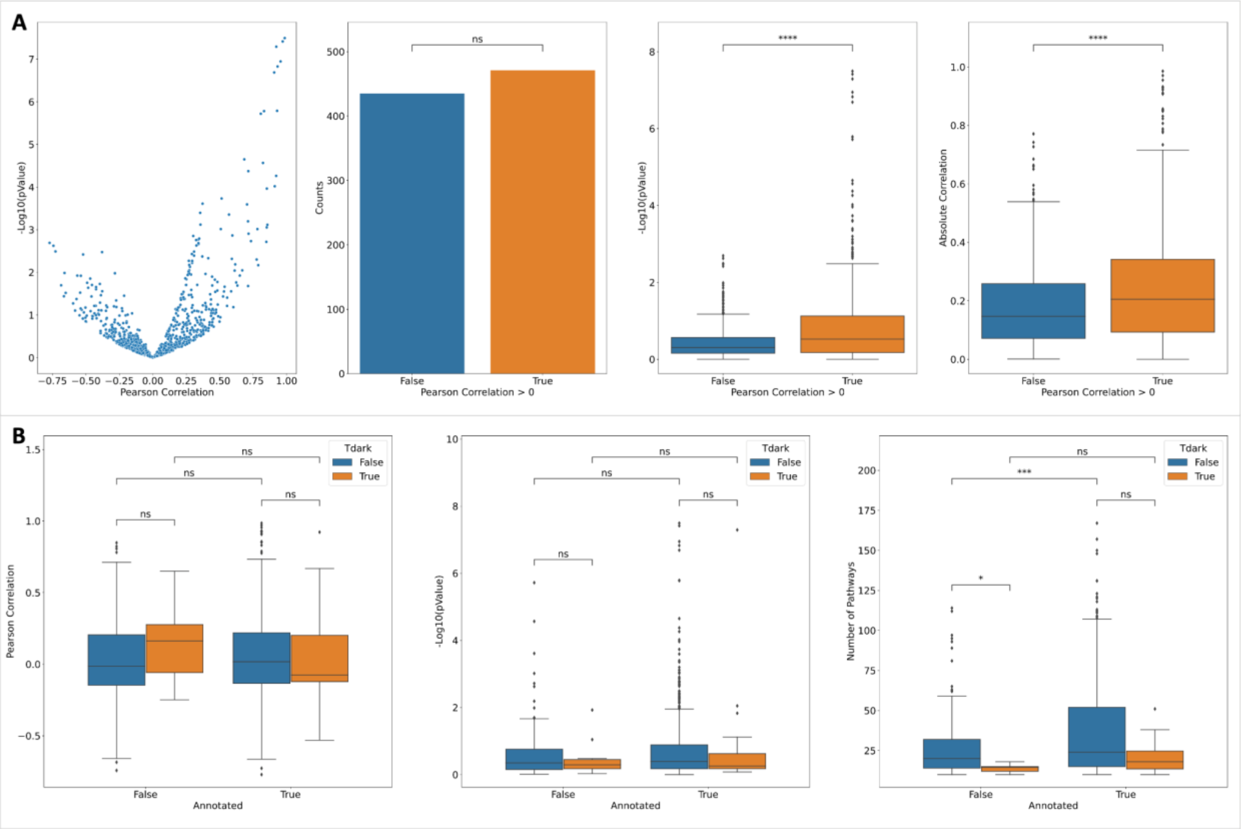

Figure S4. scRNA-seq analysis results support predicted interacting pathways by showing a significantly positively skewed distribution of correlations between average_inhibition_scores based on predicted FIs and enrichment score based on scRNA-seq coexpression (A) and unbiased distributions between annotated and not-annotated dark and not-dark proteins (B). The left-most panel in B shows the numbers of interacting pathways used for correlation calculation for individual proteins. The numbers of pathways used for correlation calculation between average_inhibition and enrichment score based on scRNA-seq co-expression are smaller than ones shown in Figure 4. This is because some proteins may functionally interact with proteins annotated in pathways but fail to have quantitative impact on pathway activities, according to our simulation approach. No significant differences were observed between the number of proteins having negative correlations and the number of proteins having positive correlations (second panel in A). Additionally, there was no significant difference in the numbers of interacting pathways for dark and non-dark Reactome annotated proteins (rightmost panel in B). Those are presumably because only gene pairs with co-expression are in the top 0.1% were selected from the scRNA-seq dataset. P-value: ****: <= 1.0E-04, ***: 1.00e-04 < p <= 1.00e-03, **: 1.00e-03 < p <= 1.00e-02, *: 1.00e-02 < p <= 5.00e-02, ns: p <= 1.00e+00.

bioRxiv preprint doi: https://doi.org/10.1101/2023.06.05.543335; this version posted June 5, 2023. The copyright holder for this preprint (which was not certified by peer review) is the author/funder, who has granted bioRxiv a license to display the preprint in perpetuity. It is made available under aCC-BY 4.0 International license.

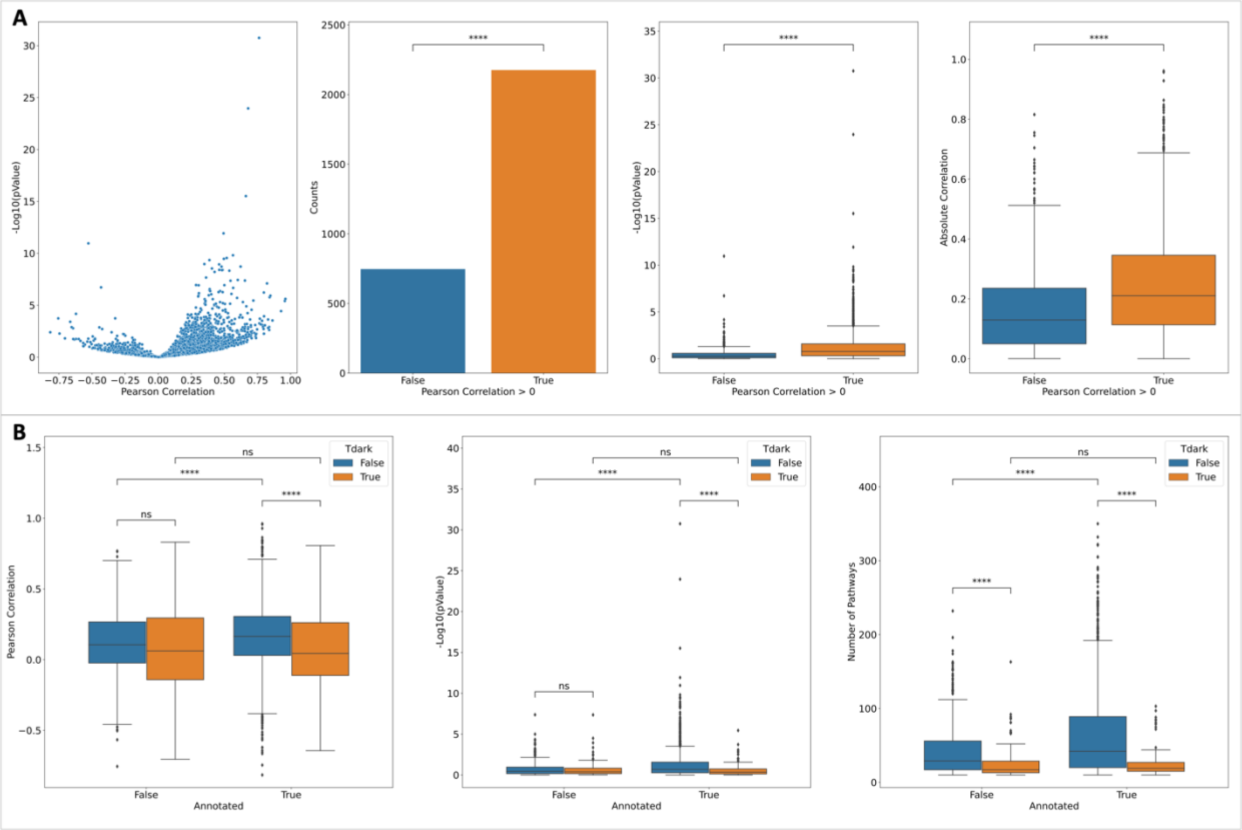

Figure S5. BERT-based NLP analysis results support predicted interacting pathways for proteins by showing a significantly positively skewed distribution. A: The distribution of Pearson correlations between NLP-based annotation scores and predicted FI-based average activation scores exhibits a significantly positively skewed distribution. **B**: The correlation difference analysis for annotated and not-annotated dark and not-dark proteins. The right-most panel in B shows the numbers of interacting pathways used for correlation calculation for individual proteins. P-value: ****: <= 1.0E-04, ***: 1.00e-04 < p <= 1.00e-03, **: 1.00e-03 < p <= 1.00e-02, *: 1.00e-02 < p <= 5.00e-02, ns: p <= 1.00e+00.

bioRxiv preprint doi: https://doi.org/10.1101/2023.06.05.543335; this version posted June 5, 2023. The copyright holder for this preprint (which was not certified by peer review) is the author/funder, who has granted bioRxiv a license to display the preprint in perpetuity. It is made available under aCC-BY 4.0 International license.

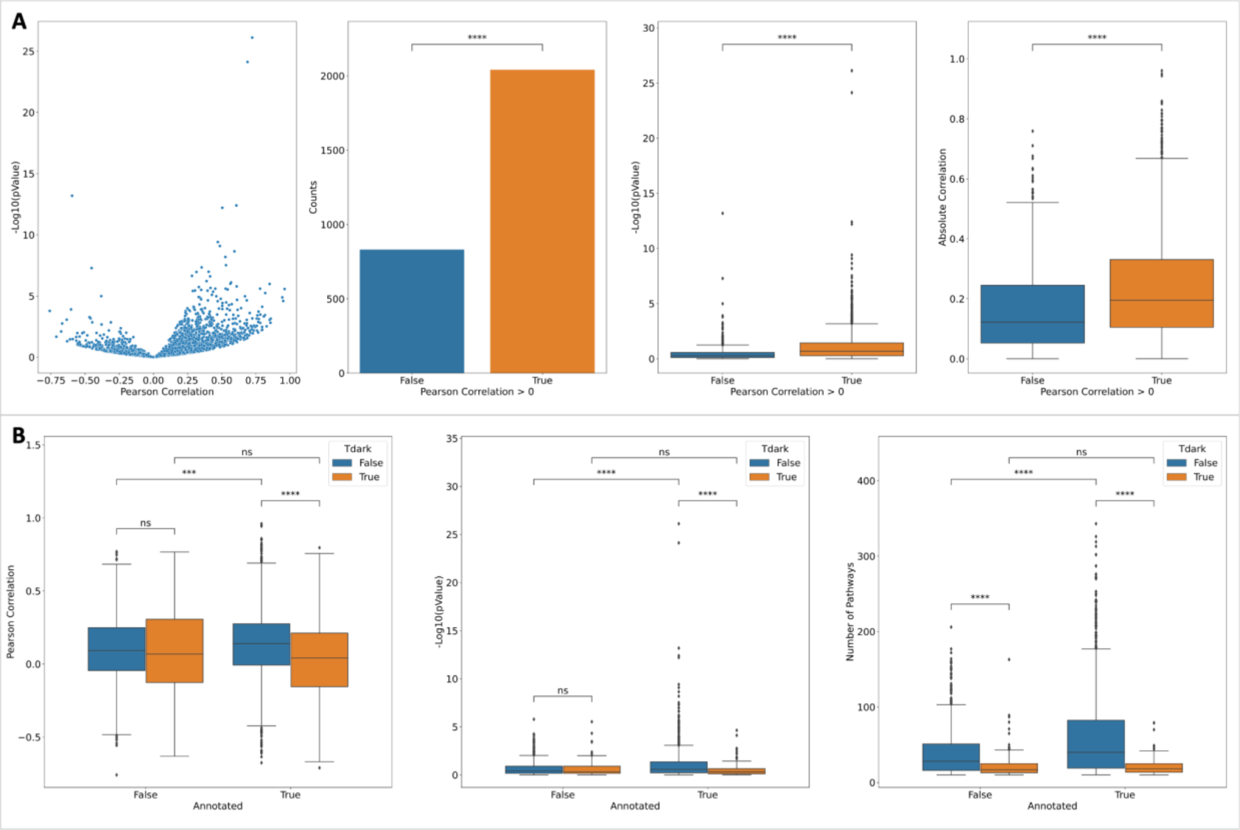

Figure S6. BERT-based NLP analysis results support predicted interacting pathways for proteins by showing a significantly positively skewed distribution. A: The distribution of Pearson correlations between NLP-based annotation scores and predicted FI-based average inhibition scores exhibits a significantly positively skewed distribution. **B**: The correlation difference analysis for annotated and not-annotated dark and not-dark proteins. The right-most panel in B shows the numbers of interacting pathways used for correlation calculation for individual proteins. P-value: ****: <= 1.0E-04, ***: 1.00e-04 < p <= 1.00e-03, **: 1.00e-03 < p <= 1.00e-02, *: 1.00e-02 < p <= 5.00e-02, ns: p <= 1.00e+00.
